# Development of a stable antibody production system utilizing an Hspa5 promoter in CHO cells

**DOI:** 10.1038/s41598-022-11342-1

**Published:** 2022-05-24

**Authors:** Hiroki Tanemura, Kenji Masuda, Takeshi Okumura, Eri Takagi, Daisuke Kajihara, Hirofumi Kakihara, Koichi Nonaka, Ryo Ushioda

**Affiliations:** 1 Biologics Technology Research Laboratories Biologics Division, Daiichi Sankyo Co., Ltd., 2716-1, Aza Kurakake, Oaza Akaiwa, Chiyoda-machi, Oura-gun, Gunma 370-0503 Japan; 2 Faculty of Life Sciences, Kyoto Sangyo University, Motoyama, Kamigamo, Kita-ku, Kyoto City, 603-8555 Japan; 3 Institute for Protein Dynamics, Kyoto Sangyo University, Motoyama, Kamigamo, Kita-ku, Kyoto City, 603-8555 Japan

**Keywords:** Cell biology, Protein folding, Chaperones, Endoplasmic reticulum, Biotechnology, Expression systems

## Abstract

Chinese hamster ovary (CHO) cells are widely used for manufacturing antibody drugs. We attempted to clone a novel high-expression promoter for producing monoclonal antibodies (mAbs) based on transcriptome analysis to enhance the transcriptional abundance of mAb genes. The efficacy of conventional promoters such as CMV and hEF1α decrease in the latter phase of fed-batch cell culture. To overcome this, we screened genes whose expression was maintained or increased throughout the culture period. Since CHO cells have diverse genetic expression depending on the selected clone and culture medium, transcriptome analysis was performed on multiple clones and culture media anticipated to be used in mAb manufacturing. We thus acquired the Hspa5 promoter as a novel high-expression promoter, which uniquely enables mAb productivity per cell to improve late in the culture period. Productivity also improved for various IgG subclasses under Hspa5 promoter control, indicating this promoter’s potential universal value for mAb production. Finally, it was suggested that mAb production with this promoter is correlated with the transcription levels of endoplasmic reticulum stress-related genes. Therefore, mAb production utilizing the Hspa5 promoter might be a new method for maintaining protein homeostasis and achieving stable expression of introduced mAb genes during fed-batch culture.

## Introduction

In the last decade, the development of monoclonal antibodies (mAbs), including immunoglobulin G (IgG), as typical biopharmaceuticals has gained increasing attention as they are promising drugs with high specificity and efficacy. More than 120 products of this type have been launched since Muromonab-CD3 was approved in 1986^1^. Chinese hamster ovary (CHO) cells are the most widely used cells for the manufacturing of antibody drugs, since they have the unique advantages of robust cell growth, effective post-translational modification, and well-established standards of good manufacturing practice (GMP) compared with other mammalian cells^2,3^.

CHO cells have been studied in various approaches to achieve high and stable production. At the genomic level, substantial effort has been made to improve expression by optimizing the genomic location of mAb-expressing vectors and increasing the copy number^4^. At the transcriptional level, higher expression has been achieved by improving promoters and introducing enhancers^5^, such as by developing a core promoter, DNA sequences (*cis*-elements), nearby promoter elements (TATA-box, CpG island, CCAAT-box, and GC-box), enhancers, silencers, and insulators^6^. In addition, various approaches have been developed to increase translational efficiency and enhance the efficiency of folding and secretion by introducing chaperones^7^. Moreover, various studies to establish optimal culture conditions have been carried out, such as by improving the culture medium^8^, aeration and stirring^9^, and pH control^10^. Furthermore, multi-omics approaches such as genomic, transcriptomic, proteomic, and metabolomic analyses have been applied to characterize CHO cells to achieve enhanced productivity^11^. In many of the above attempts, a fed-batch culture mode has been used to manufacture mAbs with developed and established CHO expression systems.

In this study, we focused on promoter activity to further increase IgG production in CHO expression systems. Various promoters have been applied in such systems, such as virus-derived promoters including those of human cytomegalovirus (hCMV), simian virus 40 early (SV40E), and Rous sarcoma virus (RSV)^5^. As heterogeneous promoters, mouse phosphoglycerate kinase 1 (PGK), human ubiquitin C (UBC), and human elongation factor-1α (hEF1α) promoters have also been used. CHO-derived promoters have also been developed, including Chinese hamster elongation factor-1α promoter (CHEF1α)^12^, along with the introduction of E77, a CHO cell-derived *cis*-element, as an enhancer^13^. There are also other examples of artificial synthetic promoters, including high-expression promoters that combine CMV and sequences from *Drosophila*^14^. In addition, inducible promoters using the Tetracycline (Tet) system have also been used^15,16^. However, unsatisfactory results are often encountered, with a lower production rate at the late culture phase than at the early logarithmic growth phase. We have also experienced problems associated with a decrease in the expression of the gene of interest in the late phase of culture. To overcome these problems, we attempted to isolate a novel high-expression promoter with long-term promoter activity derived from CHO cells by transcriptome analysis. We screened for genes whose expression was maintained until the end of culture. Specifically, transcriptome analysis was performed on CHO-K1 cells (serum-free, suspended cells derived from CHO-K1: CCL-61™; ATCC, American Type Culture Collection, Manassas, VA, USA) including multiple clones and culture media that are anticipated to be used in mAb manufacturing. This approach was implemented to identify genes with high expression under various conditions because CHO cells exhibit diverse gene expression among different clones and culture media. In addition, cell culture for transcriptome analysis was conducted in a scaled-down model with stirred-culture vessels to identify genes that are highly expressed under conditions resembling those under at-scale manufacturing. Here, it is reported that we identified a novel promoter for mAb manufacturing, optimized the promoter sequence, and assessed the productivity of various mAb sequences (IgG1, 2, 4Pro)^17–19^ to confirm that it could be generally utilized in manufacturing processes for high level of antibody production.

## Results

### Screening of highly expressed genes in transcriptome analysis

**Figure 1:**
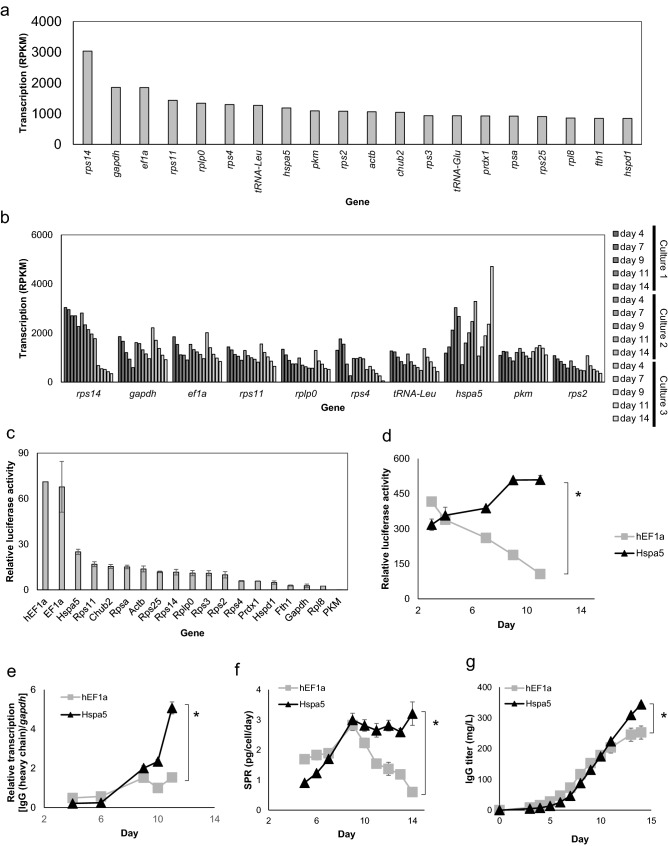
Transcriptome analysis and evaluation of the Hspa5p. (**a**) Rank of gene transcription abundance (RPKM). Three cell cultures for transcriptome analysis were performed with the following cell and medium combinations: culture 1: clone 1/G13 medium, culture 2: clone 1/CD DA1 medium, and culture 3: clone 2/G13 medium. Gene transcription was ranked by that on day 4 of culture 1, and the top 20 most highly expressed genes are described. (**b**) Gene transcription abundance (RPKM) of the three cultures on days 4, 7, 9, 11, and 14. (**c**) Luciferase assay of high-expression candidate promoters in transient expression cell pools (n = 2). (**d**) Culture time course of luciferase expression levels of stably expressing cell pools. *P < 0.05 by *t*-test on day 12 (n = 2). (**e**) Culture time course of gene expression of IgG (heavy chain). Relative transcription level was calculated using the *gapdh* gene as a control. *P < 0.05 by *t*-test on day 12 (n = 2). (**f**) Culture time course of IgG production per cell (SPR). *P < 0.05 by *t*-test on day 14 (n = 2). (**g**) Culture time course of mAb yield (IgG titer). *P < 0.05 by *t*-test on day 14 (n = 2).

To analyze the transcription level in various culture conditions, two clones of an IgG1-producing strain (CHO-K1) were cultured using custom medium G13 or CD DA1 (Supplementary Fig. S1a,b). The mRNAs were extracted from the retrieved cells throughout the culture, and comprehensive data on the transcription level of each gene were acquired by transcriptome analysis. To identify genes that were highly expressed from an earlier phase to the end of culture, transcript abundance (RPKM) was ranked using the level on culture day 4 (clone 1 and G13 medium), and the top 20 most highly expressed genes were identified, as shown in Table 1 and Fig. 1a. Among the identified genes, *gapdh* and *actβ* are known as housekeeping genes, while *ef1α* is utilized as high-expression promoters for mAb production. It was found that most of the genes identified as being highly expressed showed decreases in transcription level over the course of the culture period, while in contrast the expression of the *hspa5* [heat shock protein family A (Hsp70) member 5] gene increased (Fig. 1b, Supplementary Fig. S1c).

**Table 1:** Highly expressed genes in transcriptome analysis.

RPKM rank	Gene	Product	Database reference
1	*rps14*	Ribosomal protein S14	Gene ID:100689292/Genbank:NM_001244519.1
2	*gapdh*	Glyceraldehyde-3-phosphate dehydrogenase	Gene ID:100736557/Genbank:NM_001244854.1
3	*ef1α*	Eukaryotic translation elongation factor 1 alpha 1	Gene ID:100689276/Genbank:NM_001244402.1
4	*rps11*	40S ribosomal protein S11-like	Gene ID:100773922/Genbank:XM_003508652.1
5	*rplp0*	60S ribosomal protein lateral stalk subunit P0	Gene ID:100756201/Genbank:XM_003495916.1
6	–	tRNA-Leu	
7	*rps4*	Ribosomal protein S4	Gene ID:100689408/Genbank:NM_001246673.1
8	*hspa5*	Heat shock protein 5	Gene ID:100689305/Genbank:NM_001246739.1
9	*pkm*	Pyruvate kinase M1/2	Gene ID:100751347/Genbank:XM_003498918.1
10	*rps2*	Ribosomal protein S2	Gene ID:100689058/Genbank:NM_001244043.1
11	*actβ*	Actin beta	Gene ID:100689477/Genbank:NM_001244575.1
12	*chub2*	Polyubiquitin	Gene ID:100689267/Genbank:NM_001244378.1
13	*rps3*	40S ribosomal protein S3a-like	Gene ID:100762337/Genbank:XM_003504173.1
14	–	tRNA-Glu	
15	*prdx1*	Peroxiredoxin 1	Gene ID:100689332/Genbank:NM_001246765.1
16	*rpsa*	Ribosomal protein SA	Gene ID:100689045/Genbank:NM_001244033.1
17	*rps25*	40S ribosomal protein S25-like	Gene ID:100759466/Genbank:XM_003511566.1
18	*rpl8*	60S ribosomal protein L8-like	Gene ID:100753709/Genbank:XM_003515662.1
19	*fth1*	Ferritin heavy chain 1	Gene ID:100689102/Genbank:XM_007617280.1
20	*hspd1*	Heat shock protein family D (Hsp60) member 1	Gene ID:100689473/Genbank:XM_003504341.1

Gene transcription was ranked by RPKM for cells on day 4 in culture 1 in Fig. 1.

### The promoter of Hspa5 improves mAb expression in the latter period of fed-batch culture

The 3-kb upstream flanking region of each of the top 20 genes (Table 1) was evaluated for its function as a promoter by a reporter assay using firefly luciferase in the pGL4.10 vector (Supplementary Table S1a). Promoter activities were evaluated by transient expression of luciferase in CHO-K1 cells. As a control, hEFlα promoter (hEFlαp), which has been used as a high-expression promoter in CHO cells, was used. The results showed that higher activity was achieved for hEF1αp and Hspa5p than for other genes (Fig. 1c). Hspa5p was further investigated as a novel high-expression promoter candidate to increase mAb production because transcriptome analysis showed that the *hspa5* gene had the unique feature of being more highly expressed during the latter period of culture (Fig. 1b).

Promoter activities in stably expressing cell pools were evaluated. Luciferase activity and expression level were analyzed during the culture period. The results showed that luciferase activity and expression level under the control of Hspa5p were higher than those with hEF1αp (Fig. 1d). This is consistent with the transcriptome analysis results in which Hspa5 mRNA level increased dramatically in the latter phase of culture.

To confirm that Hspa5p can be utilized for mAb production, we constructed cells stably expressing mAb (IgG1), both heavy and light chains, under the control of Hspa5p. hEF1αp was also used as a control. To assess mAb productivity, gene expression, protein productivity per cell (specific production rate, SPR), and IgG titer were investigated. It was shown that mAb gene transcription and SPR were maintained until the latter period of culture, consistent with the luciferase assay data under the control of Hspa5p (Fig. 1e,f), while cell growth was similar (Supplementary Fig. S2). It was also shown that mAb yield on day 14 of culture was improved compared with that of the control (Fig. 1g). These findings indicate that Hspa5p improves mAb expression at the transcriptional level and can improve mAb yield as a result.

### Identification of essential Hspa5 promoter region

**Figure 2:**
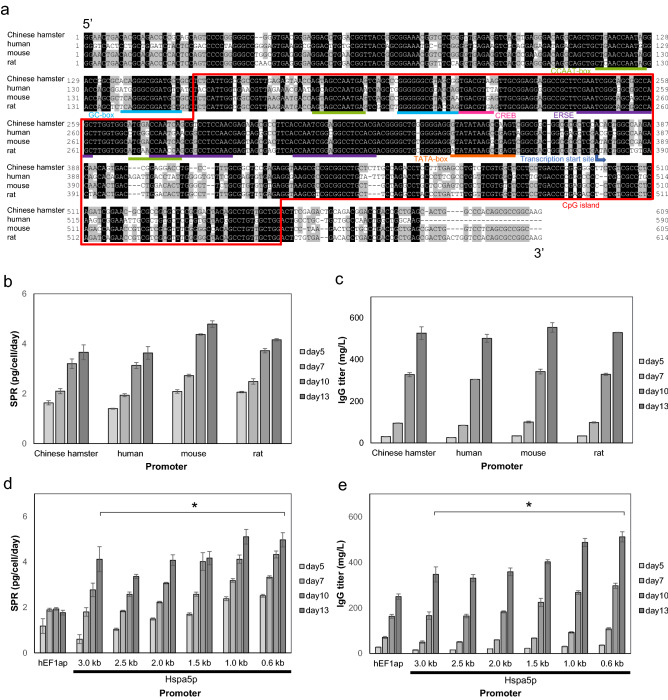
In silico analysis and characterization of Hspa5p sequence. (**a**) In silico analysis of promoter sequence of Hspa5p (~ 0.6 kb). Conservation of Hspa5p among Chinese hamster, human, mouse, and rat is shown. Core promoter elements (TATA-box, CpG island, CCAAT-box, and GC-box), cAMP-responsive elements (CREB), and ER stress-responsive elements (ERSE) are described. Transcription start site was identified by mRNA sequence from NCBI database (NM_001246739.2). (**b**,**c**) Culture time course of stable pools on days 5, 7, 10, and 13 of SPR (b) and IgG titer (**c**) with different species of heterogeneous Hspa5p (n = 2). (**d**,**e**) Culture time course of stable pools on days 5, 7, 10, and 13 of SPR (**d**) and IgG titer (**e**) with different lengths of Hspa5p. *P < 0.05 by *t*-test on day 13 (n = 2).

Detailed promoter analysis was performed to utilize Hspa5p in efficient mAb production. First, we investigated the conservation of the *hspa5* gene upstream region using GENETYX software. In general, it is suggested that consensus sequences include transcription factor binding regions. Comparison of the *hspa5* gene upstream regions of different species (Chinese hamster, human, mouse, and rat) revealed a consensus sequence approximately 0.6 kb upstream of the gene (Fig. 2a) and also found that core promoter elements, TATA-box, CpG island, CCAAT-box, and GC-box, were present in the conserved region. Furthermore, ER stress-response elements (ERSE), transcription factor binding regions of Hspa5p^20,21^, were contained in this region. We also found consensus sequences around 2.0–2.5 kb upstream, but BLAST searches did not find high homology between other functional sequences and this area (Supplementary Fig. S3).

To optimize the promoter sequence and enhance mAb production, mAb productivity was evaluated using heterologous Hspa5p. Upstream regions of the *hspa5* gene (1.0 kb) of Chinese hamster, human, mouse, and rat were cloned and studied, and mAb productivity was shown to be comparable to that with Chinese hamster-derived Hspa5p (Fig. 2b,c). This suggested that the promoter elements of *hspa5* genes were included within the regions conserved among different species. Considering that the levels of mAb production were similar, Hspa5p derived from CHO cells was used for further investigation.

Since 0.6 kb upstream of *hspa5* was suggested to be a transcriptionally functional region, truncation of the cloned region was carried out from 3.0 kb to about 0.6 kb to identify the minimum promoter length still demonstrating mAb production. *hspa5* gene upstream regions (3.0, 2.5, 2.0, 1.5, 1.0, and 0.6 kb) were used to assess mAb production. The highest productivity was achieved from 0.6 kb Hspa5p (Fig. 2d,e). Although in silico analysis showed that the consensus sequence was also around the 2.0–2.5 kb upstream region, this area did not appear to contribute to the transcriptional activity of Hspa5p (Supplementary Fig. S3). Further truncation of Hspa5p resulted in a lower gene expression level in previous studies^22–24^. Therefore, the region from the start codon of *hspa5* to 0.6 kb upstream of it was identified as the minimum promoter region of Hspa5p.

### Productivity evaluation of various IgG subclasses

**Figure 3:**
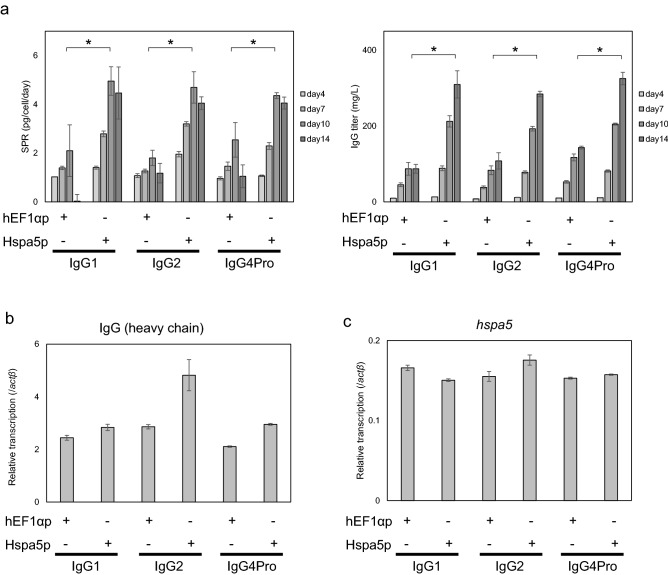
Evaluation of Hspa5p for different cell lines and IgG subclasses. (**a**) Culture time course of stable pools on days 4, 7, 10, and 14 of SPR and IgG titer with different IgG subclasses. As a promoter, hEF1αp and Hspa5p (~ 0.6 kb) were used. Regarding IgG subclasses, IgG1, IgG2, and IgG4Pro were used. *P < 0.05 by *t*-test on day 10 for SPR and on day 14 for IgG titer (n = 3). (**b**,**c**) Transcription of IgG (heavy chain) gene (**b**) and endogenous *hspa5* gene (**c**) on culture day 7. Relative transcription level was calculated using *actβ* gene as a control.

To examine whether Hspa5p can be used generally for antibody–drug production, various subclasses, IgG1, IgG2, and IgG4Pro, which have different disulfide bond patterns, were expressed in CHO-K1. This increased the productivity per cell during the latter period of culture and increased product yields (IgG titer) for each IgG subclass, in comparison to those in control conditions (hEF1αp) (Fig. 3a), while profiles of cell growth, metabolism, and product quality of mAb were similar (Supplementary Figs. S4, S5). Quantitative PCR experiments showed that the transcription level of the IgG gene under the control of Hspa5p was higher than that of hEF1αp for each IgG subclass (IgG1, IgG2, and IgG4Pro) (Fig. 3b). However, no differences were found in the expression levels of endogenous *hspa5* genes in the cells producing the different IgG subclasses (Fig. 3c), suggesting that Hspa5p is highly expressed regardless of the mAb sequence.

### Correlation between mAb production under the control of Hspa5p and UPR gene expression

**Figure 4:**
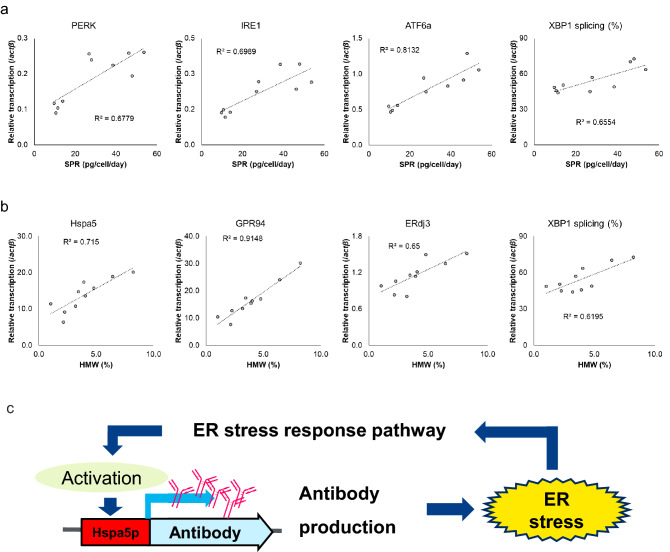
Relationship between Hspa5p productivity and ER stress-related genes. (**a**) Correlation between mAb productivity (SPR) and ER stress-related genes in Hspa5p clonal cell lines. Relative transcription level was calculated using *actβ* gene as a control. The correlation was evaluated using the correlation coefficient (R^2^). (**b**) Correlation between mAb aggregation [detected as high-molecular-weight species (HMW) by HPLC] and ER stress-related genes in Hspa5p clonal cell lines. Relative transcription level was calculated using *actβ* gene as a control. The correlation was evaluated using the correlation coefficient (R^2^). (**c**) Diagram showing the hypothesized mechanism of Hspa5p activation for mAb production.

Hspa5 is an endoplasmic reticulum (ER) chaperone activated in the unprocessed protein response (UPR), so Hspa5p is considered to be affected by UPR^25^. To assess the relationship between mAb productivity under Hspa5p and UPR gene expression, five clones of mAb-expressing CHO cells that showed different mAb productivity were cultured, and the transcription levels of the *hspa5* gene and various UPR-related genes on culture days 7 and 10 were investigated. Interestingly, the results showed that mAb productivity and mAb aggregate content were correlated with the transcription levels of some UPR genes. Regarding mAb productivity, correlations with the levels of gene transcription of PERK, IRE1, and ATF6, which are related to various ER stress-response pathways, were observed (Fig. 4a, Supplementary Fig. S6a). Correlations between the levels of mAb aggregates and ER chaperones such as GRP94 and ERdj3 were also observed (Fig. 4b, Supplementary Fig. S6b). Furthermore, the XBP1 mRNA splicing rate activated in the IRE1 pathway showed correlations with mAb productivity and aggregates. These results suggest that mAb accumulation in the ER induces ER stress, and that Hspa5p is activated by UPR (Fig. 4c).

## Discussion

In this study, transcriptome analysis was carried out for combinations of clones and media to obtain a novel effective promoter in CHO cells, considering the possibility that the level of gene transcription differs depending on the combination of cell clone and medium. In addition, by analyzing gene expression until the end of the culture, *hspa5* was identified as a gene candidate for which the transcription level improves in the latter period of culture, even though its expression in the early stage of culture was not the highest among the candidate genes. This highlights the importance of analyzing the transcription level under culture conditions resembling those in mAb manufacturing to obtain a high-expression promoter. Housekeeping genes, such as ribosomal RNA genes, *gapdh, actβ*, and *chub2*, were identified from the transcriptome analysis as highly expressed genes (Fig. 1a). Among these genes, Pkm is a kinase involved in sugar metabolism, while Prdx-1 functions in suppressing oxidative stress caused by reactive oxygen species. In addition, Hspd1 is a chaperone that functions in the mitochondria, while Fth1 is involved in cellular iron metabolism^26–30^. Fth1 showed markedly high expression in this study (Supplementary Fig. S1c), but its expression level varied across clones and media, which is not a desirable feature for mAb manufacturing. These genes, Pkm, Prdx-1, Hspd1, Fth1, are involved in basic metabolism, so they are also considered to be highly expressed housekeeping genes. Promoters of these genes were not assessed for mAb productivity​ due to their lower activity in the luciferase assay (Fig. 1c).

The identified gene of interest, *hspa5*, encodes an ER stress-response chaperone, Hspa5, which is also known as BiP or GRP78, and is involved in UPR^31,32^. UPR is a protein quality control mechanism in cells activated upon the expression of abnormal proteins, which is associated with diverse pathways such as apoptosis. Eukaryotic UPR includes four mechanisms: translational attenuation, improvement of protein folding by enhanced ER chaperone expression, abnormal protein degradation by enhanced expression of ER-associated protein degradation (ERAD)-related factors, and the induction of apoptosis. These are regulated by multiple regulatory pathways involving PERK, ATF6, and IRE1^25^. Hspa5 is globally involved in each pathway and the expression of Hspa5p is considered to be affected by UPR.

Several reports about Hspa5 proteins and promoters in CHO cells have been published. For example, Kamal et al. (2015) reported on a comprehensive analysis of UPR-related genes in clones with high and low mAb productivity, which revealed that the transcriptional activity of UPR factors including *hspa5* was enhanced in clones with high mAb productivity^33^. In addition, Pybus et al. (2014) showed that the co-expression of ER chaperones and UPR-related factors (Hspa5, ATF6c, and XBP1-s) increased mAb productivity and decreased cell proliferation^34^. An example of the use of Hspa5 promoters was also reported by Kober et al., who applied Hspa5p for productive cell clone selection^35^. However, although reports have been published on the improvement of mAb productivity by Hspa5 chaperone overexpression and the use of Hspa5p for cell clone selection, to the best of our knowledge there have been no reports on the use of Hspa5p for mAb production. Hspa5p is a novel high-production promoter with the unique feature of its associated expression being enhanced in the latter period of fed-batch culture.

Molecular mechanisms for the activation of Hspa5 expression involve transcription factors, such as ATF4, ATF6, and XBP1, and conserved elements in the Hspa5p sequence, such as CCAAT boxes, cAMP-responsive elements (CREB), and ER stress-responsive elements (ERSE)^20,21^. In silico analysis in this study also showed that the transcription factor binding regions of Hspa5p were highly conserved among the species. This may explain why there were no significant differences in mAb productivity among Hspa5p of different species. We also examined the consensus region over 0.6 kb upstream of *hspa5* because consensus sequences often contain genes or transcription factor binding regions. We found a highly conserved region 2.0–2.5 kb upstream, but no genes with high homology were identified and deletion of this region improved promoter activity, suggesting that this region does not contain a gene or transcription factor binding region activating Hspa5p (Supplementary Fig. S3).

Each IgG subclass exhibits different disulfide bonding, which might affect the activity of disulfide isomerases, such as protein disulfide isomerase-A1 (PDI)^36^. PDI is known to activate PERK pathway in UPR, thereby affecting Hspa5p activity. Therefore, we considered that differences in the folding of various subclasses, IgG1, IgG2, and IgG4Pro, might affect ER stress response and Hspa5p activity. However, differences in the structure of the subclasses used in this study did not alter mAb productivity upon the application of Hspa5p and endogenous *hspa5* gene transcription. These results suggest that the difference of disulfide bond pattern in IgG does not change the ER stress of protein folding nor UPR, including *hspa5* expression.

To consider the reason for the high expression of Hspa5p, we investigated the factors relating to Hspa5p production. Interestingly, correlations were observed between the expression of UPR-related factors and mAb productivity (Fig. 4a,b). mAb overexpression is responsible for ER stress, so it is reasonable that this correlates with the UPR. These results suggested a model in which Hspa5p activity is enhanced by the positive feedback control by the ER stress exerted by mAb production, as shown in Fig. 4c. ER stress by the overexpression of mAb and mitigation of ER stress by ER chaperone activation may be well balanced and contribute to higher production of mAb by Hspa5p application in this expression system.

On the other hand, the correlation between Hspa5p mAb productivity​ and endogenous *hspa5* gene expression was weaker than the correlations for other genes (Supplementary Fig. S6a). Their expression was not strongly correlated despite them being expressed from the same promoter, suggesting that the regulatory mechanisms may change depending on the protein expressed by Hspa5p. This result may provide novel insight into the mechanism regulating *hspa5* gene expression.

Finally, we discuss whether there is potential for further enhancement of IgG production by applying Hspa5p. Since Hspa5 is involved in many diseases such as cancer, it has been suggested as a target for drug discovery, and many reports on its transcriptional control have been published^25^. Although the expression of Hspa5 is maintained at lower levels in cells, it is considerably increased under stresses affecting ER and calcium homeostasis. Conditions enhancing the expression of Hspa5 include glucose depletion, inhibition of the protein secretion pathway such as by the addition of a calcium-depleting agent (thapsigargin), and addition of the glycosylation inhibitor tunicamycin. Furthermore, as reducing agents that disrupt disulfide bonds critical for the folding of secretory proteins, dithiothreitol (DTT) and 2-mercaptoethanol are also known to induce ER stress and enhance Hspa5 expression.

To evaluate the possibility of further enhancement of mAb productivity under Hspa5p, it was tested whether the productivity was improved upon the addition of an ER stress inducer. The results showed that, although the transcription level of the endogenous *hspa5* gene significantly increased, the transcription level of the mAb gene expressed under Hspa5p was not improved (Supplementary Fig. S7). This indicates that the induction of Hspa5p by an ER stress inducer without improving the protein-folding capacity is not effective for mAb production under Hspa5p. In addition, excessive mAb expression negatively affects protein maturation and disrupts protein homeostasis in the ER. ER stress is known to cause apoptosis, and mAb-producing cells are likely to die selectively upon ER stress. By using Hspa5 promoters, mAb production may be synchronized with the induction of molecular chaperones and oxidative isomerases to maintain protein-folding capacity in the ER, protect cells from ER stress-induced death, and thereby enable stable mAb production needed for manufacturing throughout the passages and the culture phases of a fed-batch.

## Methods

### Plasmid vector construction

As luciferase reporter and control vectors, pGL4.10 and pGL4.74 (Promega Biosciences Inc., San Luis Obispo, CA, USA) were used, respectively. pGL4.10 contains the firefly luciferase reporter gene *luc2,* and each candidate promoter was inserted into a multiple cloning site upstream of *luc2*. pGL4.74 contains HSV-TK promoter and the *Renilla* luciferase gene *hRluc.*

For mAb expression, pEF1/myc-His B (ThermoFisher Scientific, Waltham, MA, USA) was used as the backbone vector, and mAb expression genes (both heavy chain and light chain) were inserted. (Supplementary Fig. S8).

### Culture of CHO cells for transcriptome analysis

Two strains of CHO-K1 (CCL-61; ATCC, Manassas, VA, USA)^37^ expressing mAb (IgG1) were cultured in custom medium G13 (Fujifilm Wako Pure Chemical, Osaka, Japan) and CD DA1 (ThermoFisher Scientific) in a 1 L animal cell culture vessel (ABLE Corporation, Tokyo, Japan). Cultures were performed for 14 days and 1 × 10^6^ viable cells were acquired on culture days 4, 7, 9, 11, and 14, washed (300 rpm, 5 min) with PBS, and stored at − 80 °C after freezing with liquid nitrogen.

### RNA extraction and transcriptome analysis

Total RNA was extracted from thawed cells using RNAiso Plus (Takara Bio Inc., Shiga, Japan). After RNA quality analysis using Nanodrop (ThermoFisher Scientific) and Agilent 2100 Bioanalyzer (Agilent Technologies, Santa Clara, CA, USA), a sequence library was prepared using TruSeq RNA Sample Prep Kit v2 (Illumina, San Diego, CA, USA) and an automated device (Agilent Technologies). PolyA + RNAs were isolated, fragmented, and cDNA was synthesized. After blunting and phosphorylation of both ends of the synthesized cDNA, 3′-dA protrusion treatment was performed and the adaptors were ligated. DNA was amplified by PCR using a double-stranded cDNA with an adaptor as a template. Then, the PCR product obtained by the magnetic bead method using AMPure XP (Beckman Coulter, Marseille, France) was purified to generate a sequence library. High-throughput sequencing analysis was performed with the HiSeq system (Illumina) using a sequence library. A cluster that served as a template of the sequence was produced and the base sequence (fastq format) of the template DNA was acquired. The lead sequences obtained by sequence analysis were mapped to the genome sequences. The gene expression level (RPKM) normalized based on positional information was calculated.

### Transient expression of luciferase in CHO cells

A culture containing 5 × 10^5^ host CHO cells was centrifuged (240 g, 24 °C for 3 min), after which the medium was removed. Then, the cell pellet was suspended in 2 mL of Opti-MEM medium (ThermoFisher Scientific). After removing the supernatant by centrifugation, the remainder was resuspended in 2 mL of Opti-MEM medium, and 1 mL of the culture was dispensed into two wells of a 24-well culture plate. Mixtures of 3.2 µg of expression vectors and 68 µL of Opti-Pro SFM medium (ThermoFisher Scientific) were mixed with a mixture of 8 µL of gene transfer reagents and 68 µL of Opti-Pro SFM medium and reacted at room temperature for 20 min. Half of the reactants were added to each of the dispensed two wells and incubated statically in a CO_2_ incubator under conditions of 5% CO_2_ and 37 °C.

### Luciferase assay

After vector transfection, cells were cultured overnight and then the whole culture was centrifuged (9000 g, 5 min) to obtain cell pellets. Cells were washed with PBS and 100 µL of 1 × lysis buffer was added, followed by incubation at room temperature for 5 min. Dual-Luciferase® Reporter Assay System (Promega Biosciences Inc) was used for the luciferase assay. The prepared cell lysate was diluted 100-fold with distilled water and 20 µL of the diluted solution and 100 µL of Luciferase Assay Reagent II were mixed. Using this sample, firefly luciferase activity (reporter) was measured with a luminometer (OT245-01; Berthold Technologies, Bad Wildbad, Germany) for 10 s. Subsequently, 100 µL of Stop & Glo® Reagent was added, and *Renilla* luciferase activity (internal control) was measured for 10 s with a luminometer. The result of firefly luciferase measurement was divided by that of *Renilla* luciferase measurement to calculate luciferase activity.

### Construction of stable expression pools for luciferase and mAbs

After transfection and cultivation for 24 h, two wells of culture medium were collected into a single tube. The culture medium was centrifuged (240 g*,* 24 °C for 3 min) to remove the supernatant, after which the cell pellet was suspended in 4 mL of transfection medium containing 800 µg/mL Geneticin. Subsequently, this suspension was transferred to one well of a six-well culture plate, followed by incubation in a CO_2_ incubator (37 °C, 5% CO_2_) to initiate selection with Geneticin. Media were regularly replaced with transfection medium containing Geneticin (final concentration of 800 µg/mL) and expanded to T-25 culture flasks using selective expansion medium with Geneticin (final concentration of 800 µg/mL) in accordance with the growth of the cells. After incubation for 3–4 days, the culture was expanded to a 125 mL Erlenmeyer flask and used as a stable pool.

### Acquisition of monoclonal cell lines

To 100 mL of semi-solid medium was added cell culture medium diluted 100-fold with C/E medium^38^ to a viable cell density of 48 cells/mL. The mixture was gently mixed so that bubbles did not form, and the stable pool was seeded to approximately 2 mL/well on a six-well plate for a semi-solid medium. The cells were centrifuged at 240 g for 5 min at 4 °C and then cultured for 14 days in a CO_2_ incubator at 37 °C and 5% CO_2_. The colonies were picked with an automated animal cell colony picking system (Molecular Devices, LLC, Sunnyvale, CA, USA). Each picked colony was seeded into a 96-well plate for cell culture, in which 150 µL of C/E medium was dispensed into each well and cultured in the CO_2_ incubator at 37 °C and 5% CO_2_. After passage, monoclonal cells were evaluated in the fed-batch culture.

### Fed-batch culture

Stable pools and monoclonal strains expressing luciferase and mAb were evaluated by fed-batch culture in a 125 mL Erlenmeyer flask or a 15 mL small culture reactor (Sartorius, Göttingen, Germany). Production cultures in the Erlenmeyer flasks were carried out for 14 days in 5% CO_2_ at 37 °C and 120 rpm. Cultures in the 15 mL small culture reactors were conducted for 14 days at 37 °C and 850 rpm, with 50% air saturation of dissolved oxygen at pH7.00 ± 0.05 (controlled by CO_2_ and Na_2_CO_3_). Custom medium G13 and custom feed medium F13 were used. Cell concentration and viability were measured using Vi-CELL (Beckman Coulter). Metabolites were analyzed using Bio Profile FLEX2 (Nova Biomedical, Waltham, MA, USA). Antibody concentrations were analyzed by HPLC (Agilent Technologies) with a protein A affinity column (PA ID Sensor Cartridge Φ2.1 mm × 30 mm; ThermoFisher Scientific). mAb aggregation was detected by HPLC with a size-exclusion chromatography column (ACQUITY UPLC BEH200, SEC, 1.7 μm, φ4.6 mm × 300 mm; Waters, Milford, MA, USA) as high-molecular-weight species. For transcriptional analysis, cell pellets containing 1 × 10^6^ cells were acquired during the culture period and cryopreserved at − 80 °C.

### RNA extraction and reverse transcription

RNeasy Micro Kit (Qiagen, Hilden, Germany) was used for RNA extraction from cultured cells (1 × 10^6^ cells) and PrimeScript™ RT-PCR Kit (Takara Bio) was used for reverse transcription. One microliter of dNTP mixture (10 mM each dNTP), 1 µL of random 6-mers (20 µM), 1 µL of template RNA, and 7 µL of RNase-free dH_2_O were mixed and annealed using a thermal cycler at 65 °C for 5 min. Ten microliters of the reaction solution was mixed with 4 µL of 5 × PrimeScript Buffer, 0.5 µL of RNase Inhibitor, 0.5 µL of PrimeScript RTase, and 5 µL of RNase-free dH_2_O, and the reverse-transcription reaction was performed at 42 °C for 30 min, followed by 95 °C for 5 min, and the resulting cDNA solution was collected.

### Quantitative PCR

ddPCR™ EvaGreen Supermix (Bio-Rad, Berkeley, CA, USA) was used for quantitative PCR. A total of 2.2 µL of cDNA diluted in distilled water, 11 µL of 2 × ddPCR Evagreen Supermix, 1.1 µL of Forward Primer (5 µL), 1.1 µL of Reverse Primer (5 µL), and 6.6 µL of water were mixed, and droplets were made from this mixture using an Automated Droplet Generator (Bio-Rad). Subsequently, the PCR reaction was carried out under the conditions of 95 °C for 5 min, 40 cycles of 95 °C for 30 s and 62 °C for 1 min, 4 °C for 5 min, and 90 °C for 5 min. cDNA copy numbers amplified using Droplet Reader were quantified. The housekeeping genes *gapdh* and *actβ* were used as internal controls.

### Promoter analysis in silico

The sequences of *hspa5* upstream regions and information on the transcription origin (cDNA sequences) were retrieved from the NCBI database. The software GENETYX-SV/RC Ver 13.1.1 (GENETYX, Tokyo, Japan) was used for sequence analysis. Cut-off values of Polymerase II promoter analysis were as follows: TATA-box -8.16, Cap signal -3.75, CCAAT-box -4.54, and GC-box -4.9. CpG island analysis conditions were set as follows: window size 100, average span 10, minimum CpG island length 200, minimum GC content 50%, and minimum Obs/Exp CpG 0.6. BLAST analysis was used to analyze the homology of upstream gene regions.

### Addition of ER stress inducers

CHO-K1 cell clone expressing IgG1 under Hspa5p was cultured. Then the following were added as ER stress inducers on day 7 of fed-batch culture and mAb and *hspa5* gene transcription levels were analyzed: DTT (final concentrations of 1, 2, 5, and 10 mM), tunicamycin (final concentrations of 0.01, 0.05, 0.1, 0.2, and 0.5 µg/mL), and thapsigargin (final concentrations of 0.002, 0.005, and 0.01 µg/mL). Cells for transcriptional analysis were obtained on day 10 of culture, and transcription levels of IgG and endogenous *hspa5* were analyzed.

## Supplementary Information

Supplementary Information.

## Data Availability

The datasets generated during this study are available in the Gene Expression Omnibus (GEO) (https://www.ncbi.nlm.nih.gov/geo/) under the accession number GSE197570.
