# Supplementary material for: Development of a stable antibody production system utilizing an Hspa5 promoter in CHO cells
Source: Sci Rep. 2022 May 24;12:7239. doi: 10.1038/s41598-022-11342-1 (PMC9130236; doi:10.1038/s41598-022-11342-1)
Supplement: Supplementary file 1 — Supplementary Information. [file 41598_2022_11342_MOESM1_ESM.pdf]

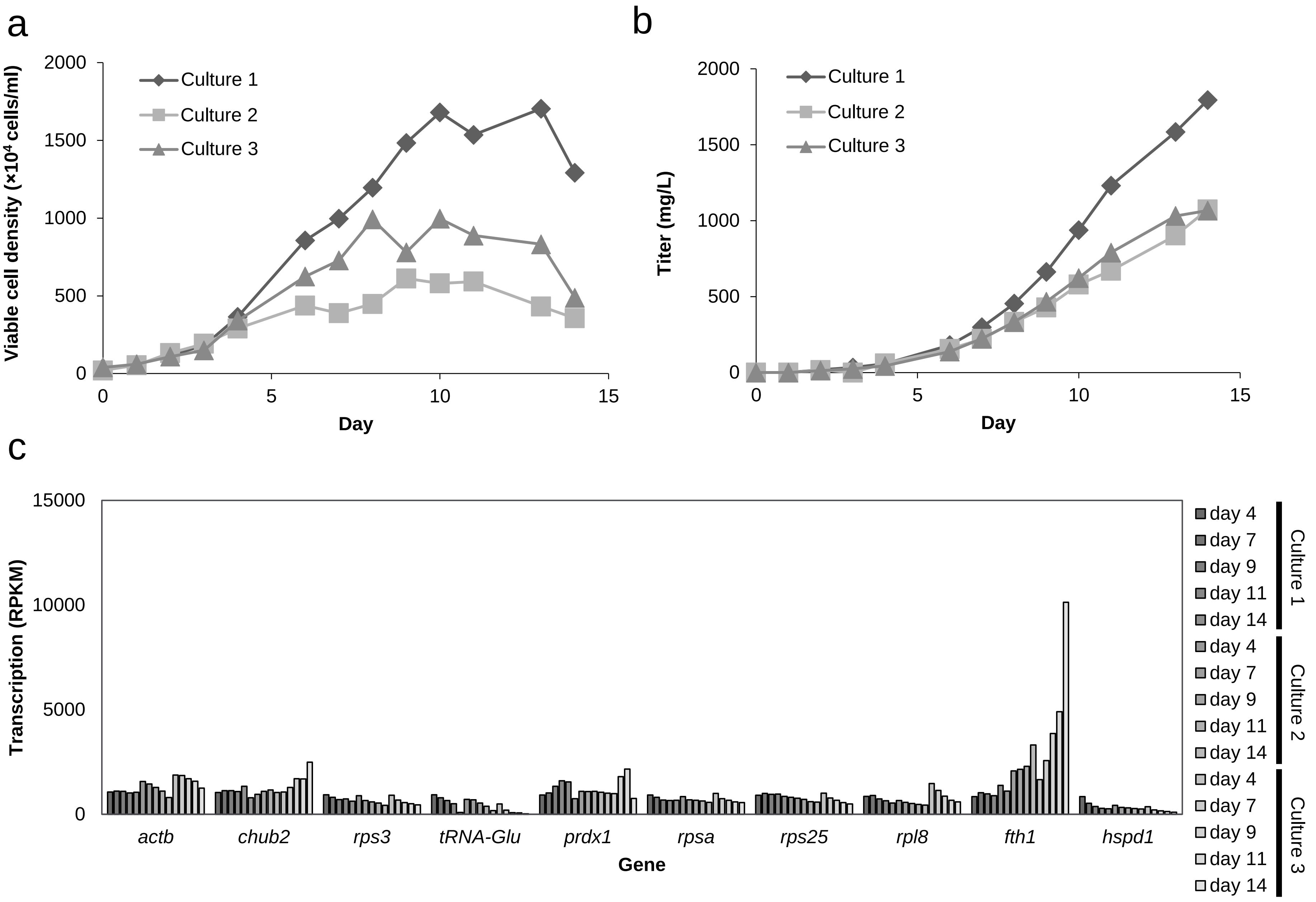

Supplementary Figure S1. Transcriptome analysis.

(a, b) Time course of cell proliferation (a) and IgG titer (b) upon transcriptome analysis. Each culture was performed with the following combinations of cell lines and media: culture 1: clone 1/G13 medium, culture 2: clone 1/CD DA1 medium, and culture 3: clone 2/G13 medium. (c) Gene transcription abundance (RPKM) of the three cultures on days 4, 7, 9, 11, and 14.

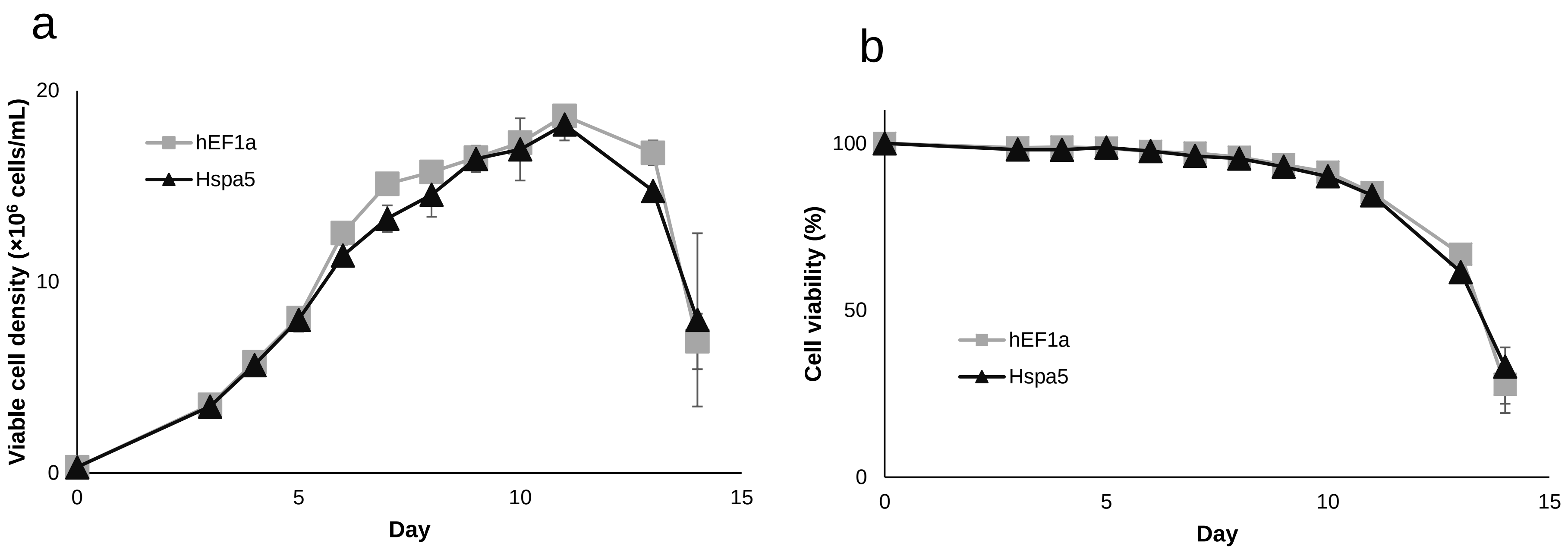

Supplementary Figure S2. Cell culture profile of fed-batch culture
Culture time course of stable pools expressing mAb (n = 2). (a) Viable cell density (b) Cell viability.

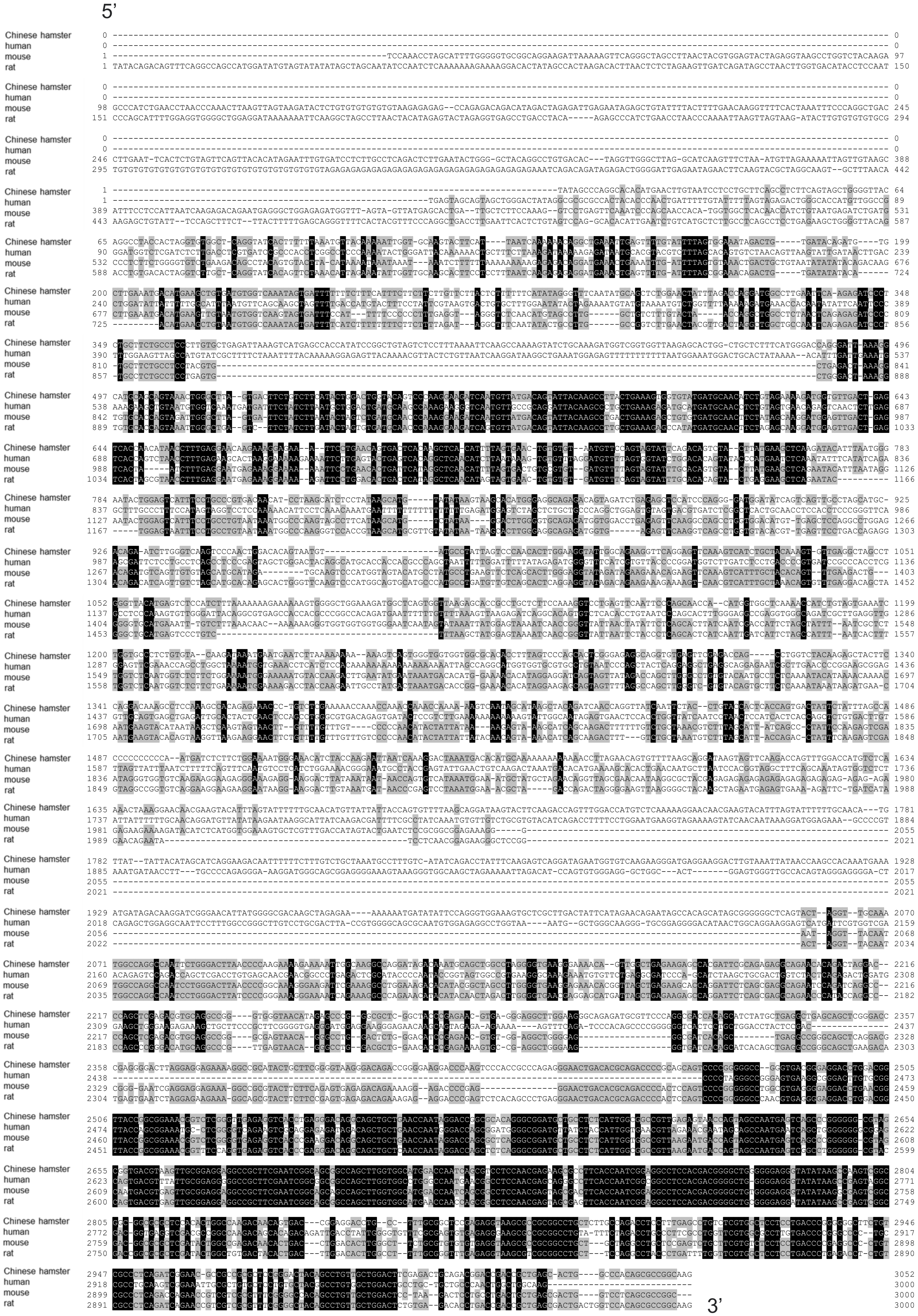

Supplementary Figure S3. Homology of heterogeneous *hspa5* gene upstream region.
Conservation of the promoter sequence of the upstream region of *hspa5* (3.0 kb) among Chinese hamster, human, mouse, and rat is shown.

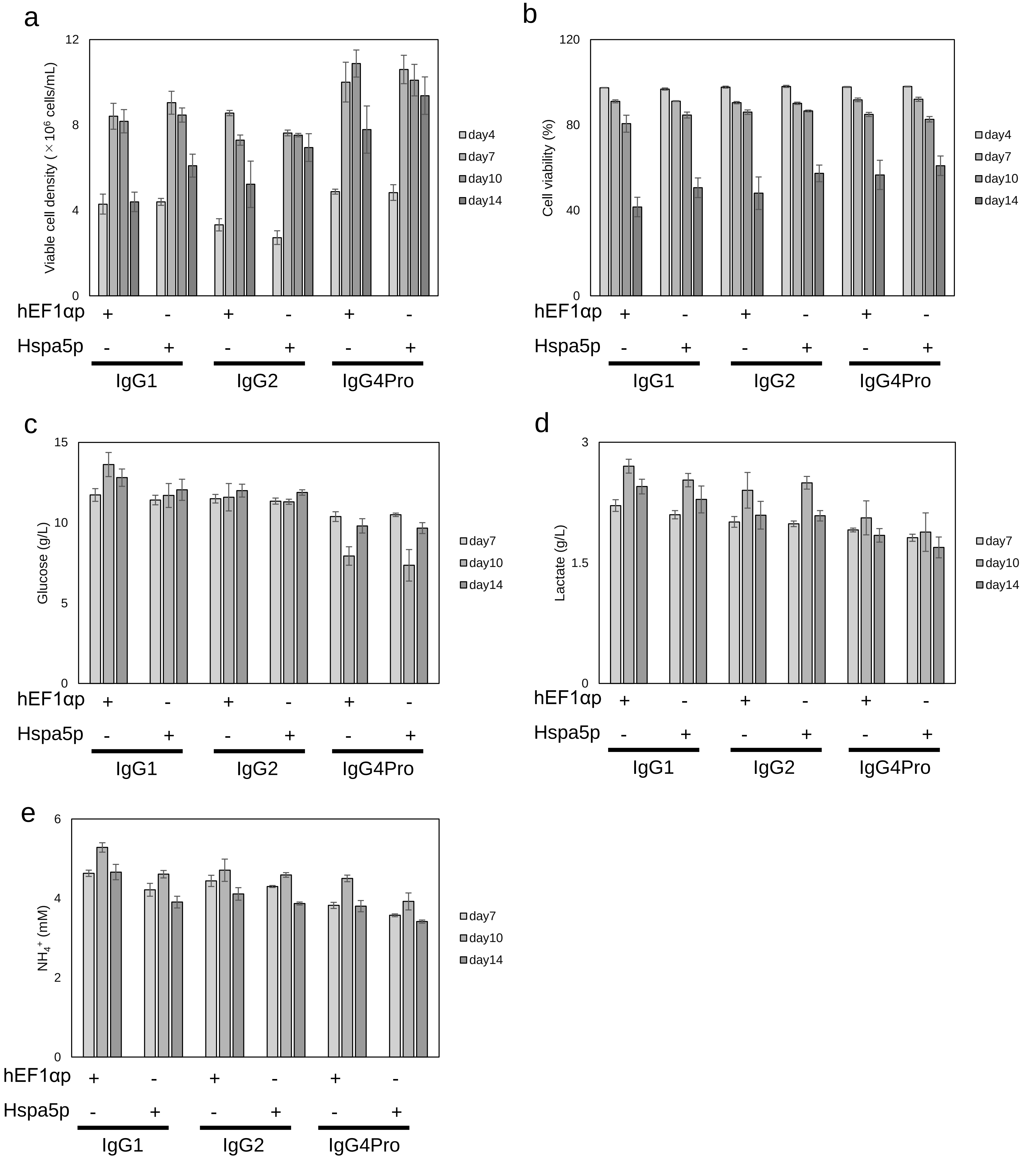

Supplementary Figure S4. Cell culture profile of fed-batch culture

Culture time course of stable pools on days 4, 7, 10, and 14 with different IgG subclasses. As a promoter, hEF1αp and Hspa5p (~0.6 kb) were used. Regarding IgG subclasses, IgG1, IgG2, and IgG4Pro were used (n = 3). (a) Viable cell density (b) Cell viability (c) Glucose concentration (d) Lactate concentration (e) NH_4^+ concentration.

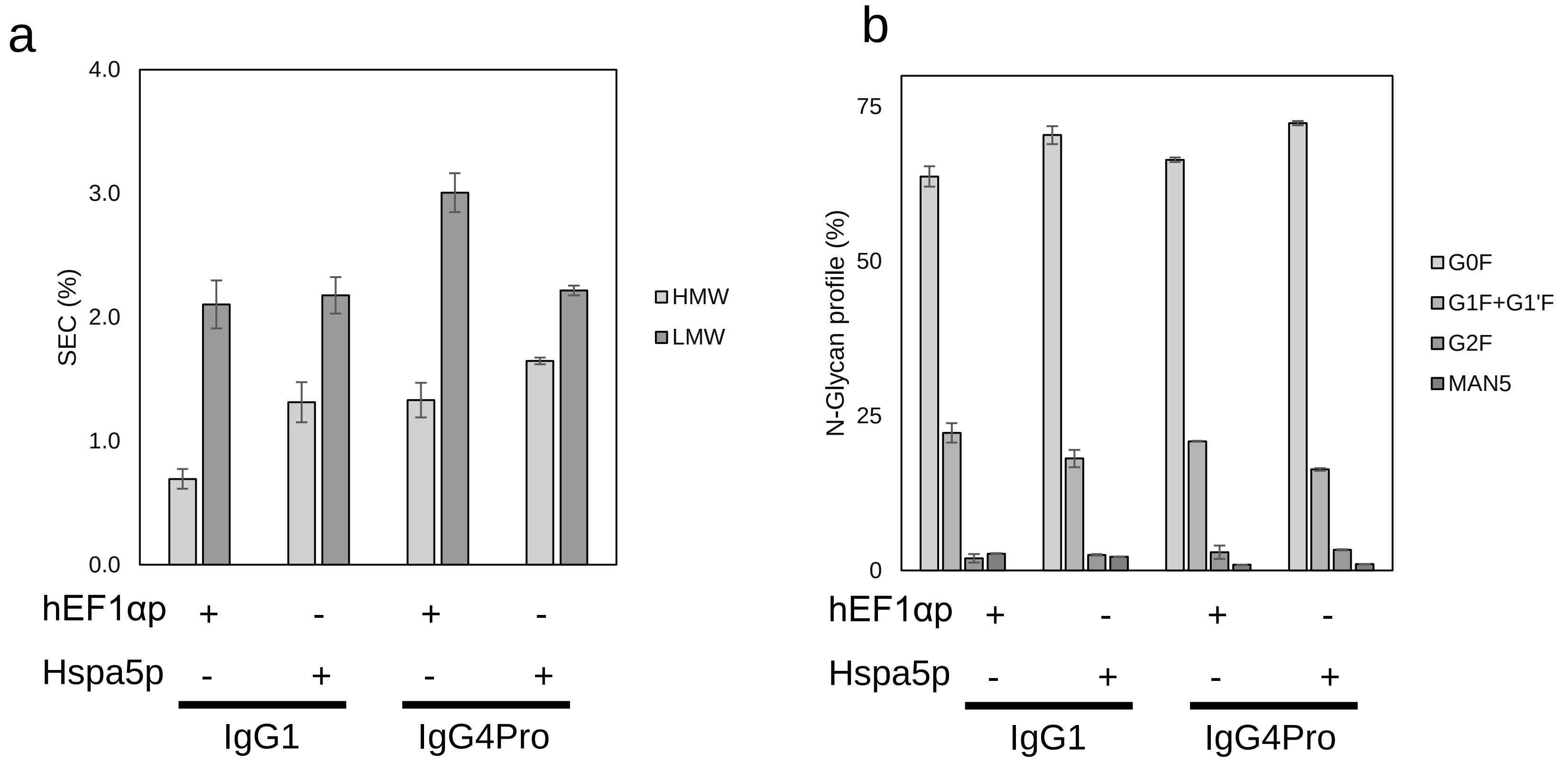

Supplementary Figure S5. Product quality of fed-batch culture

Product quality of stable pools on day 14 with different IgG subclasses (n = 3). (a) Size exclusion chromatography profile (SEC). HMW and LMW represent high molecular weight species and low molecular weight species, respectively. (b) N-Glycan profile analyzed by HPLC. For glycan purification and labeling, EZGlyco® mAb-N Kit with 2-AB (Sumitomo Bakelite Co., Ltd) was used.

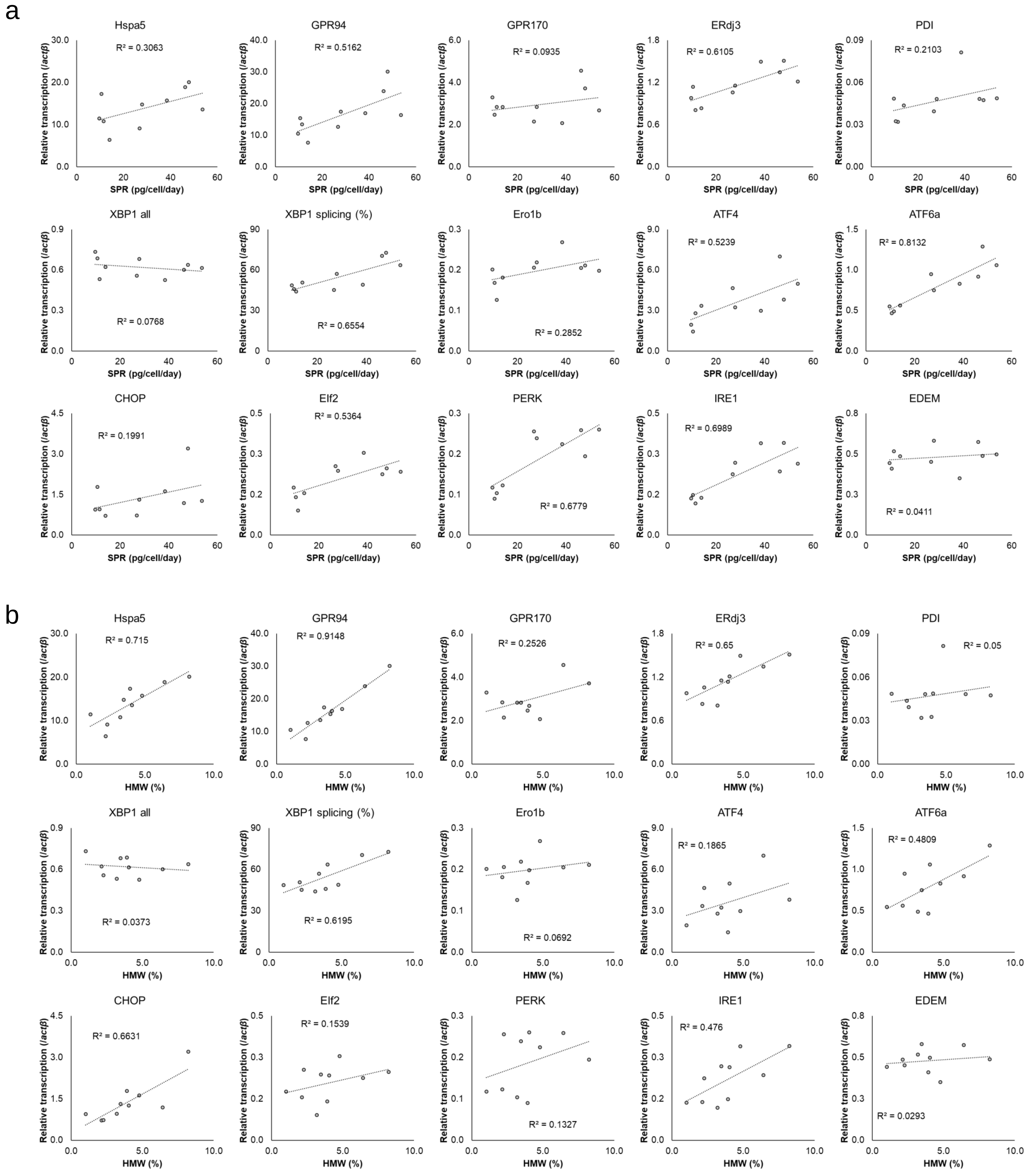

Supplementary Figure S6. Relationship between Hspa5p productivity and ER stress-related genes.
(a) Correlations between mAb productivity (SPR) and ER stress-related genes in Hspa5p monoclonal cells. Relative transcription level was calculated using the *actβ* gene as a control. The correlation was evaluated using the correlation coefficient (R^2). (b) Correlation between mAb aggregation [detected as high-molecular-weight species (HMW) by HPLC] and expression of ER stress-related genes in Hspa5p monoclonal cells. Relative transcription level was calculated using the *actβ* gene as a control. The correlation was evaluated using the correlation coefficient (R^2).

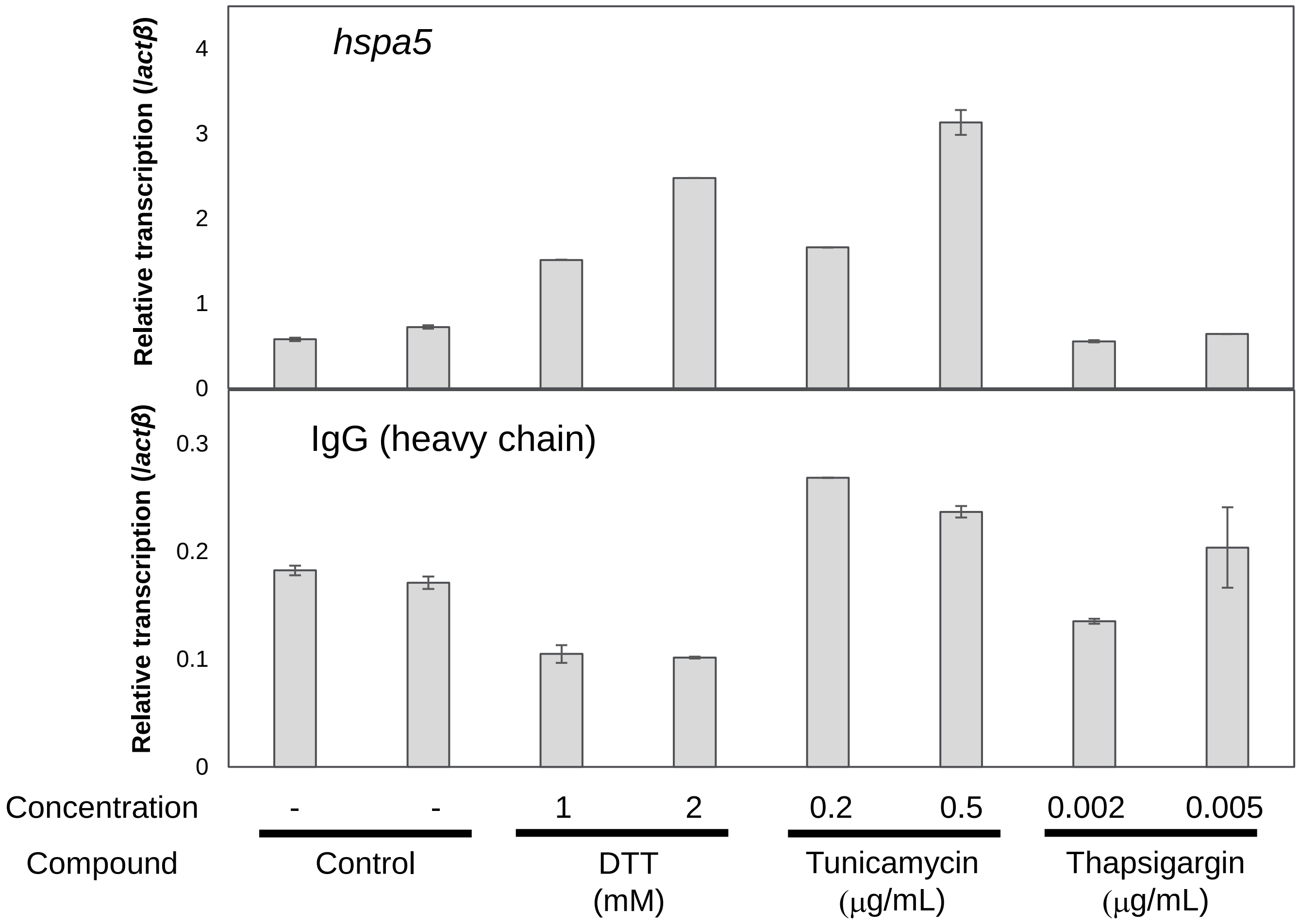

Supplementary Figure S7. Induction of ER stress by adding ER stress inducers.
CHO-K1 cell clone expressing IgG1 under Hspa5p was cultured. ER stress inducers were added on day 7 of fed-batch culture and mAb and *hspa5* gene transcription levels were analyzed. The added ER stress inducer and concentration and transcription level of *hspa5* and IgG (heavy chain) are shown. Relative transcription level was calculated using the *actβ* gene as a control.

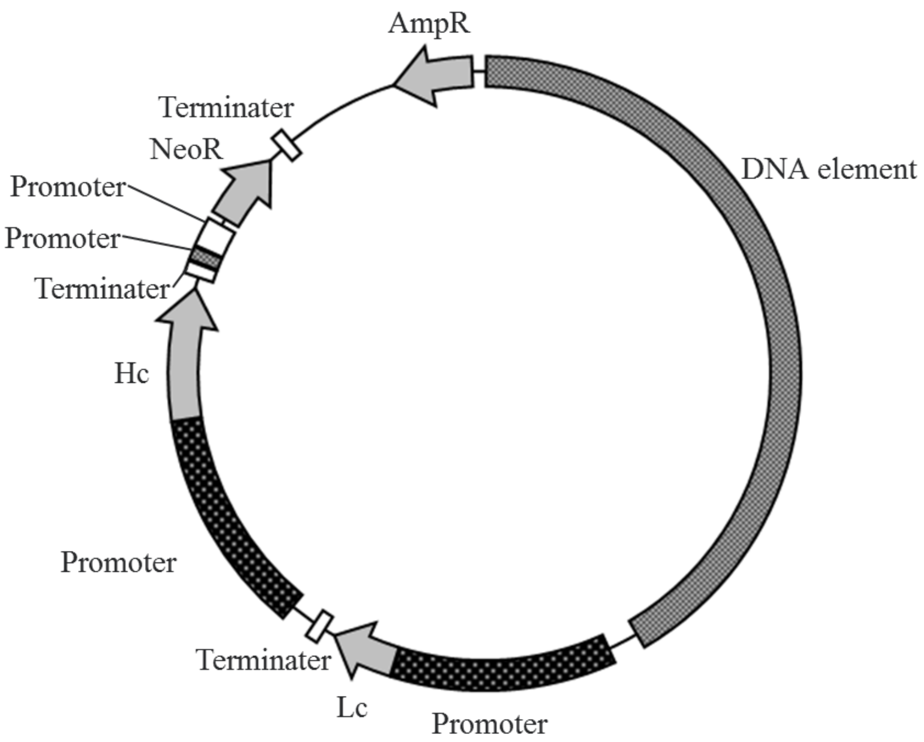

Supplementary Figure S8. Configuration of a monoclonal antibody expression vector.
pEF1/myc-His B (Life Technologies Corporation) was the backbone vector for the expression vector. Lc and Hc which are representing the genes of the light chain and heavy chain of a monoclonal antibody, respectively. The genes of the light chain and heavy chain were synthesized. DNA elements were originally cloned from human genomic DNA.

Supplementary Table S1. Primer sequences used in this study.
(a) Primers for promoter cloning. (b) Primers for quantitative PCR analysis.

a

Promoter	Restriction enzyme		Forward primer sequence	Reverse primer sequence
	Forward	Reverse		
Rps14	XhoI	HindIII	tcctcgagGGACTCAAAGCACAAGCTCA	tcaagcttTTCTGAGTGGAAGGAGAACC
Gapdh	NheI	XhoI	tcgctagcCACTCAGCAACGAAAGTCCA	tcctcgagTGCGTCTCCGGAGCGAGGCT
Eef1a1	XhoI	HindIII	tcctcgagACACAGTGAGTTTGAGGCCA	tcaagcttGTTGGATTTGAATTAGCGGT
Rps11	NheI	XhoI	tcgctagcATGATGAAGCGATCTCCCAC	tcctcgagCTTCCCGGCAGCCTGAGGAA
Rplp0	XhoI	HindIII	tcctcgagCTTCCTGGAGGTTGCAAAAG	tcaagcttCACGGCGGTGCGTCAGGGAT
Rps4	KpnI	XhoI	tcggtaccGAGAAACCGGAAAATCACCC	tcctcgagGGCTGCGCTGGGAACGGAAA
Hspa5	KpnI	HindIII	tcggtaccTATAGCCCAGGCACACATGA	tcaagcttCTTGCCGGCGCTGTGGGCCA
PKM	NheI	XhoI	tcgctagcCTCTTGTTTTTGAGCTGGGG	tcctcgagGGTTTCTGAGGTCCTGGGTC
Rps2	NheI	XhoI	tcgctagcAACTCAAGGGCAAACCTGTG	tcctcgagTTGCTAGAGAAGCAAAAAAG
Actb	NheI	HindIII	tcgctagcCCATTGCAGACCAGACAGAA	tcaagcttGGCGAACTATATCAGGGCAC
Chub2	NheI	HindIII	tcgctagcGAATGGTGGGATTGAAGGTG	tcaagcttTGTCTGTCACACACACAAAC
Rps3	XhoI	HindIII	tcctcgagGACAGGGTTTGTGGCATCTT	tcaagcttGGTGCTGCTGAGAGAGCCAA
Prdx1	XhoI	HindIII	tcctcgagGGGATTAAAGTCGTGAGCCA	tcaagcttCTTGCTATCAGCTGAAAAAG
Rpsa	KpnI	XhoI	tcggtaccTTCGGGAAGACTGGAGCTAA	tcctcgagTGTGTAAGTTTCCCTTATAA
Rps25	NheI	HindIII	tcgctagcGCCAGACCCAGAAGACAAAA	tcaagcttGATGAAGCTCGGAGAGTGGC
Rpl8	XhoI	HindIII	tcctcgagGATTTCTCTGCATTCGAGGC	tcaagcttGGCGGCGAGTCTGGAAAGGG
Fth1	XhoI	HindIII	tcctcgagGTTCCATCCCCAAGACTGAA	tcaagcttGGCGGCGGCGGGCGCGGTGG
Hspd1	KpnI	XhoI	tcggtaccAATTCTGGGTCCTTCTGCCT	tcctcgagTTCTGGGAGAGGGGGGGAAA
Hspa5 (2.5 kb)	Not1	HindIII	ggggggcggccgcTGGTCGGTGGTTAAGAGCAC	tcaagcttCTTGCCGGCGCTGTGGGCCA
Hspa5 (2.0 kb)	Not1	HindIII	ggggggcggccgcTCCCAACTGGACACAGTAAT	tcaagcttCTTGCCGGCGCTGTGGGCCA
Hspa5 (1.5 kb)	Not1	HindIII	ggggggcggccgcAATTCTACCTGTACCACTCA	tcaagcttCTTGCCGGCGCTGTGGGCCA
Hspa5 (1.0 kb)	Not1	HindIII	ggggggcggccgcCGGGAACATTATGGGGCGAC	tcaagcttCTTGCCGGCGCTGTGGGCCA
Hspa5 (0.6 kb)	Not1	HindIII	ggggggcggccgcGGAACTGACACGCAGACCCC	tcaagcttCTTGCCGGCGCTGTGGGCCA
Hspa5 (human)	Not1	NheI	gtgttgcggccgcACAGTAGGGAGGGGACTCAGAGC	gtggggctagcCTTGCCAGCCAGTTGGGCAGCAG
Hspa5 (mouse)	Not1	XbaI	ggtgggcggccgcATGGTGGAAAGTGCTCGTTTGACC	ggtggtctagaGCCGGCGCTGAGGACCAGTCGCTC
Hspa5 (rat)	Not1	XbaI	ggtgagcggccgcCTCAACGGAGAAGGGCTCCGGAC	ggtaggtctagaCTTGCCGGCGCTGTGGACCAGTC

b

Gene	Forward primer sequence	Reverse primer sequence
Actβ	GTGCTATGTTGCCCTGGACT	AAGGAAGGCTGGAAAAGAGC
Hspa5	CCAGCGCCAAGCAACCAAAG	TATCCAGGCCATACGCAATAGCAG
GPR94	ACCTGCTGCATGTCACAGAC	AAAAACTCGCTCGTTCCAGA
GPR170	TCCGTTATTTCCAGCACCTC	GTCTGCCTCTGTGGGTCAAT
ERdj3	GGAGGTGGTTTGTGACGAGT	GCCATCTCTCACACCAGGTT
PDI	CAACAGCCAGACGTCACCTA	TCTGTCAGCAGTTCCCTGTG
XBP1 spliced	ACTACTGAAGAGGCTCCAGAGACGG	GAGGTGCACGTAGTCTGAGTGCTG
XBP1 full	ACTACTGAAGAGGCTCCAGAGACGG	TGACAACTGGGCCTGCACCT
Ero1b	AAGCGACCATGTCCTTTTTG	TGCCCAGCTTTAATTCCAAC
ATF4	AGCACTTCAAACCTCATGGGTTCTC	GGTGTCTGAGGCACTGACCAAC
ATF6α	CTCCTCGCTCCGTAGACTCTTG	CTGAGGATGGGCTATTACAGCTTTC
CHOP	GGAAATGAAGAGGGGGAGTC	AGCTGCTTGTGACCTCTGCT
EIf2	TTCTGGAGCTTTCCAGCAAT	GCCCTGCTCTAATCTTGCAC
PERK	CCTGGTGGGAACAAAGAAGA	CAATCAGCAACGGAAACCTT
IRE1	CCAAACGTGATCCGCTACTT	GCAAAGTCCTTCTGCTCCAC
EDEM	TACCAGGCAACCAAGAATCC	GCATACCCGCATTTGACTTT
IgG (heavy chain)	TCCGCCTCCTTCCTGTACTC	GCTGGAGATTGTCAGGGTAAAG
IgG (light chain)	GCCAGCCCGAGAACAACTAC	CACGGTCAGCTTGGAGTACAG
